# Direction-of-Arrival Estimation Based on Frequency Difference–Wavenumber Analysis for Sparse Vertical Array Configuration

**DOI:** 10.3390/s23010337

**Published:** 2022-12-28

**Authors:** Donghyeon Kim, Gihoon Byun, Jeasoo Kim

**Affiliations:** 1 Department of Convergence Study on the Ocean Science and Technology, Korea Maritime and Ocean University, Busan 49112, Republic of Korea; 2 Department of Ocean Engineering, Korea Maritime and Ocean University, Busan 49112, Republic of Korea

**Keywords:** direction-of-arrival estimation, sparse vertical array configuration, spatial aliasing, frequency difference-wavenumber analysis

## Abstract

Frequency–wavenumber (*f*–*k*) analysis can estimate the direction of arrival (DOA) of broadband signals received on a vertical array. When the vertical array configuration is sparse, it results in an aliasing error due to spatial sampling; thus, several striation patterns can emerge in the *f*–*k* domain. This paper extends the *f*–*k* analysis to a sparse receiver-array, wherein a multitude of sidelobes prevent resolving the DOA estimates due to spatial aliasing. The frequency difference-wavenumber (Δf–*k*) analysis is developed by adopting the concept of frequency difference, and demonstrated its performance of DOA estimation to a sparse receiver array. Experimental results verify the robustness of the proposed Δf–*k* analysis in the estimation of the DOA of cracking sounds generated by the snapping shrimps, which were recorded by a sparse vertical array configuration during the shallow water experiment.

## 1. Introduction

The direction of arrival (DOA) of a signal propagated over an ocean waveguide is a primary factor in various applications and procedures, such as estimating of the location of the source and restoring the transmitted signal in a passive environment [1,2,3]. DOA estimation has recently been applied in Green’s function estimation of unknown sources (e.g., ships) [4,5,6,7]. The frequency–wavenumber (*f*–*k*) analysis, as well as reliable and robust delay-and-sum (DAS) beamforming, can estimate the DOA of a uniform linear array with *d*-m spacing [8,9,10]. In the *f*–*k* analysis, the DOA is calculated by using the ratio of the wavenumber, determined by the frequency of the signal, with the wavenumber derived by spatial sampling. If the frequency of the signal exceeds the design frequency (=c/2d, where *c* is the nominal sound speed in water of 1500 m/s), the angle would be inaccurately estimated due to spatial aliasing. A statistical approach can be used to rectify the DOA of a broadband signal in a single-path environment [9]. However, angle correction is difficult when multipaths exist, and the frequency band of the signal is significantly higher than the design frequency (i.e., sparse).

For a sparse vertical array configuration, DOA estimation by using DAS beamforming is highly likely to fail, and recent efforts have been made to overcome this problem. Abadi et al. proposed a beamforming approach based on the concept of frequency difference, known as frequency-difference (FD) beamforming [11]. FD beamforming is a method of beamforming a difference-frequency component that is equal to the difference between two frequencies extracted by the product of two relatively high-frequency components [11,12,13]. Xenaki et al. applied the concept of compression sensing (CS) to beamforming in 2014 [14,15,16,17,18]. CS-based beamforming is a method used for estimating the DOA by using a convex optimization solution under sparse vertical array configurations.

In this paper, we propose an extended *f*–*k* analysis for a sparse vertical array configuration by utilizing the FD concept of and explaining the relationship with the existing FD beamforming. Hereafter, the algorithm proposed in this paper is referred to as the frequency difference–wavenumber (Δf–*k*) analysis.

The remainder of this paper is organized as follows: in Section 2, we review the frequency–wavenumber analysis and present the mathematical formulation of the Δf–*k* analysis proposed herein. In Section 3, we describe the shallow-water acoustic variability experiment (SAVEX15) conducted in the northeastern East China Sea (ECS). Section 4 compares the *f*–*k* analysis, Δf–*k* analysis and FD beamforming via simulation for the SAVEX15 environment, demonstrating the feasibility of DOA estimation by using the Δf–*k* analysis. In Section 5, we describe the estimation of the DOAs of cracking sounds produced by snapping shrimps, which were recorded by using a sparse vertical array during the SAVEX15 experiment, using the Δf–*k* analysis and FD beamforming. Finally, the concluding remarks are summarized in Section 6.

## 2. Mathematical Formulation

### 2.1. Review of the Frequency–Wavenumber Analysis

The *f*–*k* analysis is defined as a time–space Fourier transform of the signal received from an array. Here, we review the relationship between the *f*–*k* analysis and DAS (or conventional) beamforming [9]. A simple case with a linear array of M sensors and a single-ray path is considered. In a single-path scenario, the signal received by one array element, $s(\omega_{o},r_{m})$, is given as follows:

(1) $$s\left(\omega_{o},r_{m}\right)=A\left(\omega_{o}\right)\exp\left(-i\omega_{o}r_{m}\sin\theta_{o}/c\right).$$

Here, $r_{m}$ represents the *m*th array element location and $\theta_{0}$ represents the DOA, with the positive angle representing an upgoing path. The $\omega_{o}$ and *A* denote the frequency and amplitude of the signal, respectively.

Because the exponent of the exponential term in Equation (1) can be separated into the wavenumber component on the array axis [$k_{o}=\left(\omega_{o}/c\right)\sin\theta_{o}$] and position vector, Equation (1) can be represented as follows:

(2) $$s\left(\omega_{o},r_{m}\right)=A\left(\omega_{o}\right)\exp\left(-ik_{o}r_{m}\right).$$

The beam output in a specific beam angle θ is given as

(3) $$B\left(\omega_{o},\theta \right)=\sum_{m=1}^{M}s\left(\omega_{o},r_{m}\right)\exp\left[i\omega_{o}\tau_{m}\left(\theta \right)\right],$$

where $\tau_{m}\left(\theta \right)$ is the time delay for the ray path that arrives at the *m*th receiver at nominal elevation angles θ from the horizontal plane. For simple plane-wave beamforming, $\tau_{m}\left(\theta \right)$ can be computed from

(4) $$\tau_{m}\left(\theta \right)=r_{m}\sin\theta /c,$$

where *c* is the nominal sound speed in water at 1500 m/s.

Substituting Equation (4) into Equation (3) and expressing this equation in the spatial domain using the wavenumber component [$k=(\omega_{o}/c)\sin\theta$] for the steering angle yields the following result:

(5) $$B\left(\omega_{o},\theta \right)=\sum_{m=1}^{M}s\left(\omega_{o},r_{m}\right)\exp\left(ikr_{m}\right)=B\left(\omega_{o},k\right).$$

Equation (5) is a spatially discrete Fourier transform (DFT) form of a frequency-domain signal received via an array, known as the *f*–*k* analysis. That is, the *f*–*k* analysis and DAS beamforming are closely related [9], and the peak (i.e., DOA) corresponds to the wavenumber $k_{o}=\left(\omega_{o}/c\right)\sin\theta_{o}$.

### 2.2. Physical Region of the f–k Analysis

In the case of a uniform linear array with *d*-m element spacing, which corresponds to *D* = 1 in Reference [9], the spatial DFT period is 2π/d and the wavenumber range is

(6) $$-\frac{\pi }{d}\leq k\leq \frac{\pi }{d}.$$

When the number of wavenumber bins in the spatial DFT is *N*, which is an even number, the grid of wavenumbers is given as

(7) $$k_{l}=\frac{2\pi l}{Nd}\left(l=-\frac{N}{2}+1,\cdots ,0,\cdots ,\frac{N}{2}-1,\frac{N}{2}\right).$$

Recalling the wavenumber component [$k=(\omega_{o}/c)\sin\theta$] for the steering angle, the look angle grid is given as

(8) $$\theta_{l}=\sin^{-1}\left(\frac{\lambda_{o}l}{Nd}\right)\left(l=-\frac{N}{2}+1,\cdots ,0,\cdots ,\frac{N}{2}-1,\frac{N}{2}\right).$$

Here, $\lambda_{o}=2\pi c/\omega_{o}$ and the period of the look angle is $\lambda_{o}/d$.

Because $\theta_{l}$ is a function of frequency (or wavelength), the position of the peak on the wavenumber grid varies with frequency, even at the same angle [10]. For example, if the frequency of the signal is the same as the design frequency (i.e., $\lambda_{o}=2d$) and $\theta_{o}=90^{\circ }$, a peak appears in the wavenumber grid corresponding to l=N/2. Furthermore, when the frequency of the signal is lower than the design frequency for the same angle $\theta_{o}$, $\lambda_{o}$ becomes greater than 2d, resulting in a peak in the grid lower than N/2, which is still within the wavenumber grid. In contrast to the previous two cases, $\lambda_{o}<2d$ at a frequency higher than the design frequency. Therefore, if $\theta_{o}=90^{\circ }$, the theoretical peak position determined by Equation 8 is greater than N/2 and exceeds the wavenumber grid [8].

As such, if the theoretical peak position exceeds the wavenumber grid, a peak appears on the grid that is subtracted from the theoretical wavenumber value by an integer multiple of 2π/d due to the spatial DFT period (2π/d). This wavenumber shift, known as spatial aliasing [8], results in an inaccurate DOA estimation, which is a type of angle filtering by an array. This indicates that the angle that the array can physically detect at a frequency higher than the design frequency is restricted [10]. All angles can be detected at frequencies lower than the design frequency. However, as the frequency increases above the design frequency, the range of angles that can be detected gradually decreases. In the case of DAS beamforming, forcing the output of all angles is possible, even at frequencies higher than the design frequency. However, the grating lobe causes ambiguity. Consequently, there is a tradeoff between angle restriction and the appearance of grating lobes.

In the case of a broadband signal, the peaks corresponding to a frequency band form a striation with a slope providing DOA information [10]. In a single-path environment, even if spatial aliasing occurs, the DOA may be corrected by using the statistical approach [8]. However, if a broadband signal is received through a multipath and has sufficiently high frequencies to indicate that the array configuration is sparse, the physically detectable angle range will be extremely narrow. Consequently, the angle cannot be estimated by using the *f*–*k* analysis because of striation interference. Furthermore, reaching the near field of the array is likely if the array configuration is sparse. Therefore, we proposed a time–space Fourier transform based on the FD concept to address this problem, as described in the next section.

### 2.3. Frequency Difference–Wavenumber Analysis

When the frequency band of the signal is considerably higher than the design frequency, the FD concept is employed to obtain the low-frequency component [11,12,13,19,20,21]. This is performed by taking the quadratic product of the two in-band frequency components of the received signal as follows:

(9) $$s^{\prime }\left(\bar{\omega },\Delta \omega ,r_{m}\right)=s\left(\bar{\omega }+\Delta \omega /2,r_{m}\right)s^{*}\left(\bar{\omega }-\Delta \omega /2,r_{m}\right).$$

Here, the difference frequency, Δω, is the difference between the two frequencies (i.e., $\Delta \omega =\omega_{2}-\omega_{1}\left(\omega_{2}>\omega_{1}\right)$) that must come from within the signal bandwidth. When $\Omega_{L}$ and $\Omega_{H}$ define the lower- and upper-frequency bounds of the signal bandwidth, respectively, the average frequency of the two frequencies, $\bar{\omega }=\left(\omega_{1}+\omega_{2}\right)/2$, is within the following range: $\Omega_{L}+\Delta \omega /2\leq \bar{\omega }\leq \Omega_{H}-\Delta \omega /2$. The asterisk denotes a complex conjugate. $s^{\prime }\left(\bar{\omega },\Delta \omega ,r_{m}\right)$ is a signal with the Δω component, but it may not be a version in which the frequency of the original signal is completely downconverted to Δω.

To overcome the problem that occurs in the *f*–*k* analysis when the array configuration is sparse, this paper proposes a method (called the Δf–*k* analysis) that utilizes the FD concept as a preprocessing step before performing spatial DFT.

Recalling that the *f*–*k* analysis is a two-dimensional Fourier transform of the received signal as a function of time and space, the Δf–*k* analysis is formulated by estimating the difference-frequency component from Equation (8) and performing spatial DFT as follows:

(10) $$B\left(\bar{\omega },\Delta \omega ,k\right)=\sum_{m=1}^{M}s^{\prime }\left(\bar{\omega },\Delta \omega ,r_{m}\right)\exp\left(ikr_{m}\right).$$

For *Q* total wavefront arrivals, rather than a single arrival, a quadratic product, such as Equation (9), comprises *Q* desired terms and $Q^{2}-Q$ unintended terms [19]. Although the desired terms mimic the field at the difference frequency, unintended terms with a cosine factor that varies with $\bar{\omega }$ may result in the formation of sidelobes. However, for a sufficiently high $\bar{\omega }$, the cosine factor sign changes abruptly as $\bar{\omega }$ varies, allowing the unintended terms to be suppressed by considering an incoherent average throughout the signal bandwidth [11,12,13,19,20,21]. Thus, the following signal bandwidth-averaging is necessary to mimic the *f*–*k* analysis at a low frequency from the Δf–*k* analysis:

(11) $$B\left(\Delta \omega ,k\right)={\langle {\left|B(\bar{\omega },\Delta \omega ,k)\right|}^{2}\rangle }_{\bar{\omega }}.$$

If the difference frequency is within the design frequency and spatial aliasing does not occur, the peak in the wavenumber axis of the Δf–*k* analysis calculated by using Equation (10) appears at $\Delta k_{o}=\left(\Delta \omega_{o}/c\right)\sin\theta_{o}$.

For a broadband signal, a striation can form in a frequency band that is significantly lower than the design frequency because various difference-frequency components can be obtained from the signal bandwidth. Thus, in the aforementioned multipath environment, it is feasible to separate the striations by minimizing striation interference. Additionally, the conversion to a low frequency makes the source in the near field appearto be in the far field.

We compare the results of the proposed algorithm and FD beamforming by using simulated and experimental data, respectively, as described in Section 4 and Section 5.

## 3. SAVEX15

**Figure 1:**
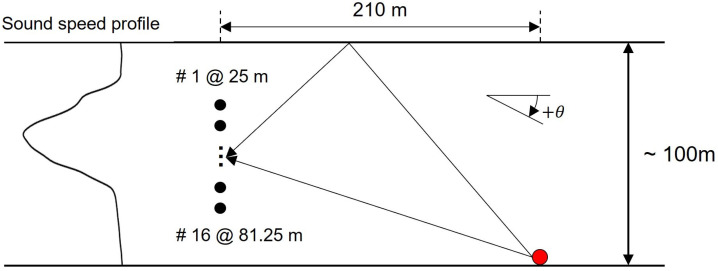
Schematic of the VLA and sound–speed structure on JD 141 during SAVEX15. The SSP is a CTD profile collected on JD 141. A VLA with 16 elements (black circles) uniformly spaced at 3.75 m apart is bottom-moored inapproximately 100-m deep water. A positive grazing angle θ is defined as an upgoing path. The range between the snapping shrimp (red circle) and the VLA is approximately 210 m.

In May 2015, SAVEX15 was conducted in the northeastern ECS by using the research vessel Onnuri [22]. Figure 1 shows a schematic of the experiment with the sound–speed profile (SSP) measured from a conductivity, temperature, and depth (CTD) profile collected on JD 141 [22]. The bottom-moored vertical line array (VLA) comprised 16 elements, with an aperture of 56.25 m and an element spacing of 3.75 m (i.e., design frequency = 200 Hz), covering about half the water column (from 25–81 m) in approximately 100-m deep water. The acoustic transmissions were in various frequency bands covering 0.5–32 kHz and included both channel-probing waveforms and communication sequences. Throughout the experiment, highly impulsive noises produced by the snapping shrimps, which usually thrive at depths of less than 60 m [23], were unexpectedly received on large-aperture vertical arrays and dominated the soundscape [24,25]. Cracking sounds, known as snaps, have the most spectral energy at higher frequencies (>10 kHz) and are composed of two arrivals (i.e., direct and surface-reflected arrivals). When ambient noise data with no acoustic transmissions and only cracking sounds were analyzed, the dominant frequency band of the cracking sounds was found to be 11–24 kHz. At the lower-frequency bound (11 kHz) of the dominant frequency band, the element spacing corresponded to 27.5 wavelengths, rendering the array configuration extremely sparse. To verify the proposed algorithm, we used the snaps recorded during the experiment as well as the simulation data by using a 60-ms cosine-tapered linear frequency modulation chirp with the same frequency band (i.e., 11–24 kHz) as snaps. The source and VLA configurations, as well as the SSP shown in Figure 1, were utilized in the simulation, and the source was assumed to be on the seabed, comparable to the snapping shrimps [24].

## 4. Numerical Simulation

The ray-tracing code BELLHOP was used to generate the received signals for the simulation [26,27]. The Green function between the source and receiver can be calculated by using the following equation:

(12) $$H_{m}\left(\omega \right)=\sum_{q=1}^{Q}a_{qm}\exp\left(i\omega t_{qm}\right),$$

where $a_{qm}$ is the arrival amplitude, including any phase shift from boundary bounces, and $t_{qm}$ is the arrival time. These two variables are the outputs of BELLHOP. The received signals were generated by multiplying the Green function with the frequency-domain transmitted signal and performing an inverse Fourier transform.

As previously mentioned, we used two ray-path arrivals (i.e., Q=2) to mimic the scenario of snapping shrimp, discussed in Section 5. The source depth and the range between the source and the VLA were set to 100 m and 210 m, respectively [24].

**Figure 2:**
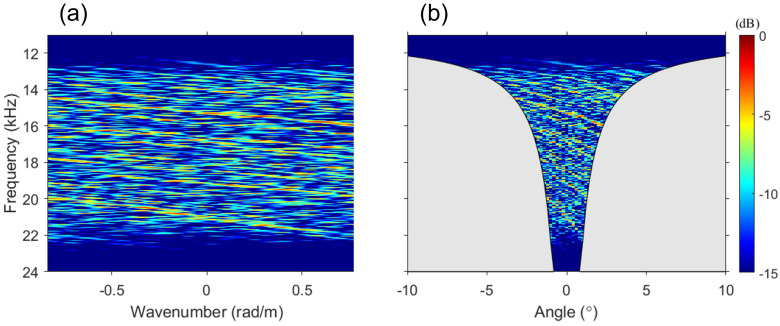
*f*–*k* analysis of the simulation data, with the geometry shown in Figure 1; (**a**) frequency–wavenumber domain, and (**b**) frequency–angle domain. A 60-ms cosine-tapered linear frequency modulation chirp in the same frequency band as that generated by the snapping shrimp is utilized for the simulation, resulting in a sparse vertical array configuration. Despite the two-path environment, the *f*–*k* analysis is featureless. Because the array configuration is sparse, the gray-shaded region, where the array cannot physically detect the DOA, is extremely wide. Thus, the DOA cannot be estimated due to striation interference by the multipath.

Figure 2a shows the *f*–*k* analysis of the simulation data. The slopes appear to be visible; however, overall, it is featureless to the extent that the angle cannot be estimated, although there were only two ray paths. This is because of the near field of the array as well as striation interference. Considering that the far field of the array is reached when $L^{2}/4\lambda r$ is less than unity [28], the source will be in the near field because this parameter at 11 kHz (the lower frequency bound) is greater than 27 (i.e., $L^{2}/4\lambda r={\left(56.25\;m\right)}^{2}/\left[4\left(0.1364\;m\right)210\;m\right]$=27.6). If the source is in the near field, the slope related to the DOA in the *f*–*k* analysis inevitably spreads, and the influence of the spread is increased in the case of a sparse vertical array configuration. With $k_{o}=\left(\omega_{o}/c\right)\sin\theta_{o}$, the x-axis was converted from a wavenumber to a physically detectable angle, as shown in Figure 2b. Recalling that the angle range that the array can detect by using Equation (8) decreases when the frequency of the signal is higher than the design frequency, the angle range becomes extremely narrow for an extremely sparse vertical array configuration, as discussed in Section 2. The gray-shaded region represents the regions that are not physically detectable, and, when the design frequency is 200 Hz, the detectable angle range of the array at 24 kHz is within $\pm 0.8^{\circ }$. Figure 2b, similar to Figure 2a, is featureless, as if the pattern of striations is random. Hence, the DOA cannot be corrected by using periodicity.

**Figure 3:**
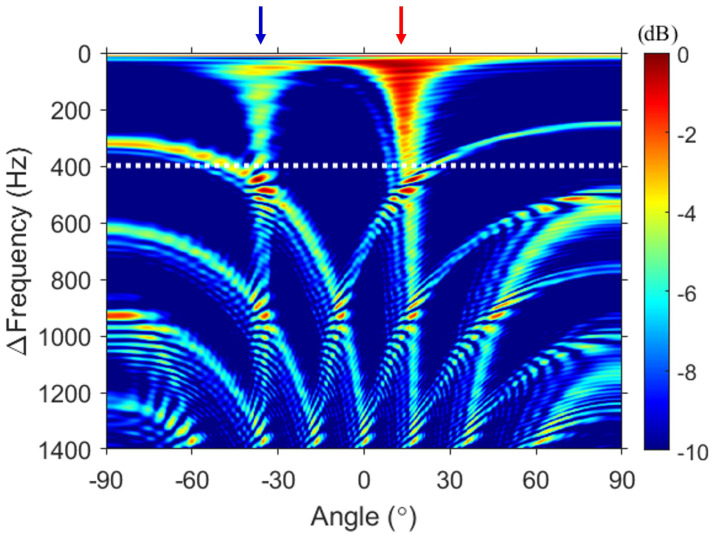
FD beamforming of the simulated data. To minimize the number of unintended terms generated due to the multipath, the FD beamforming outputs are averaged over the signal frequency band (i.e., 11–22.6 kHz), resulting in two distinct vertical lines between −60${}^{\circ }$ and 30${}^{\circ }$. The red and blue arrows indicate the DOAs corresponding to the direct and surface-reflected paths, respectively. The two arrows are aligned with the angle represented by the two vertical lines, showing that FD beamforming can estimate the DOAs of a sparse vertical array configuration.

First, FD beamforming was applied to the same simulation (see Figure 3). To minimize the number of cross terms generated due to the multipath, the output of FD beamforming was incoherently averaged over 11 kHz $\leq \omega_{1}\leq$ 22.6 kHz with 10-Hz intervals. The y-axis in Figure 3 indicates the difference frequency. To confirm the trend of an increasing difference frequency, the difference frequencies, which are user-chosen parameters, were set from 0 Hz to 1400 Hz with 1-Hz intervals. This is comparable to simulating a signal with a frequency band of 0–1400 Hz. The arrows in Figure 3 represent the DOAs calculated by using the image method based on the center of the VLA as the reference angles. The red and blue arrows correspond to the direct (12.6${}^{\circ }$) and surface-reflected (−36.1${}^{\circ }$) paths, respectively.

Two vertical lines (mainlobes) are observed in the FD beamforming output, and the angles corresponding to two vertical lines are in good agreement with the DOAs. Several curves in Figure 3 are grating lobes caused by spatial aliasing and are a mix of grating lobes produced by each path. In Figure 3, the white dotted line represents the maximum limit frequency (i.e., 400 Hz) that satisfies the far field of the array for the geometry considered here. A frequency higher than 400 Hz (lower part of the white dotted line in Figure 3) satisfies the near field of the array, resulting in sidelobes emerging around the main or grating lobes due to the angle spread.

**Figure 4:**
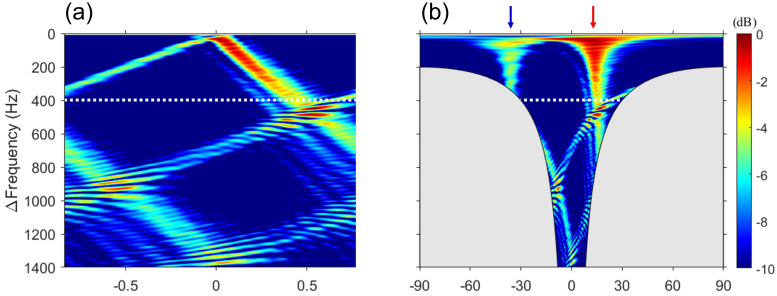
Δf–*k* analysis of the simulated data. (**a**) Difference frequency–wavenumber domain and (**b**) difference frequency–angle domain. The output of the Δf–*k* analysis is averaged similarly to that of FD beamforming. The Δf–*k* analysis and Figure 3 are identical, except for the gray-shaded region. Compared with the featureless *f*–*k* analysis, the Δf–*k* analysis clearly exhibits two lines. Considering the range between the source and VLA and aperture of the VLA, the maximum limit frequency of the far field of the array (white dotted line) is 400 Hz. Angle spread can occur because of the near-field effect on difference frequencies higher than the maximum limit frequency, as shown in (**a**,**b**).

The results of the proposed algorithm for simulation data are displayed in Figure 4a. The result of Δf–*k* analysis is incoherently averaged over 11 kHz $\leq \omega_{1}\leq$ 22.6 kHz with 10-Hz intervals, similar to that of FD beamforming. The difference between Figure 2a and Figure 4a is remarkable. In contrast to the featureless *f*–*k* analysis (see Figure 2a), the output of the Δf–*k* analysis shows that the two main gradients are clearly separated because multipath interference is mitigated.

Although spatial aliasing exists in the difference-frequency band of 0–1400 Hz, clear separation allows for the DOA estimation through periodicity. However, beyond the maximum limit frequency (white dotted line), other minor slopes are formed in addition to the major slopes, which shows the angle spread due to the near-field effect explained in FD beamforming. This angle spread can be relaxed if the range is increased, whereas other conditions remain fixed. Figure 4b shows the result of wavenumber-to-angle conversion, using $\Delta k_{o}=\left(\Delta \omega_{o}/c\right)\sin\theta_{o}$, as shown in Figure 2. The gray-shaded region represents the angle range that the array cannot detect physically. When the difference frequency is lower than the design frequency, an output at all angles was achieved, similar to that in FD beamforming. However, as the difference frequency is increased, the angle range decreases. Nevertheless, the Δf–*k* analysis can detect a wider angle compared with the *f*–*k* analysis.

**Figure 5:**
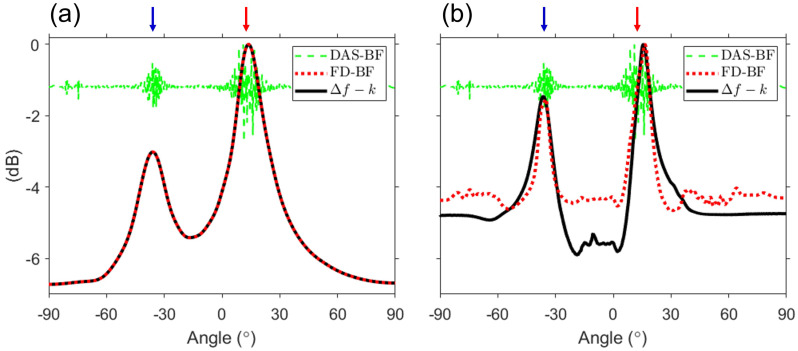
For simulation data, comparison of the Δf–*k* analysis (black solid line) with FD beamforming (red dotted line) double averaged over 0 ≤Δf≤ (a) 200 Hz (design frequency) or (b) 1400 Hz with DAS beamforming (green dashed line). For double averaging, the Δf interval is 1 Hz, and the gray-shaded region in Figure 4 is excluded. Along with FD beamforming, the Δf–*k* analysis has the ability to estimate any angle within the design frequency. By contrast, the sidelobes of the Δf–*k* analysis are lower than those of FD beamforming when averaging over all difference-frequency bands, because the grating lobes of FD beamforming have the same function as adding background noise.

Except for the gray-shaded region, Figure 3 and Figure 4b are identical. This relationship is consistent with that described in Sec. Section 2 between the *f*–*k* analysis and DAS beamforming and is corroborated by Figure 5. Additional averaging (i.e., double averaging) in the range of difference frequencies can improve the robustness of DOA estimation [11,12,19,20]. Figure 5 shows a comparison between DAS beamforming, the results of double averaging from the Δf–*k* analysis and FD beamforming. Averaging for Δf was performed with 1-Hz intervals, excluding the gray-shaded regions. The green dashed line represents DAS beamforming, whereas the black solid line and the red dotted line represent the double averaging outputs of the Δf–*k* analysis and FD beamforming, respectively. All the results are normalized to the peak value. First, Figure 5a shows the results of double averaging within the difference frequency band lower than the design frequency as well as the result of DAS beamforming.. Two large pulses are detected in DAS beamforming. However, the exact angle could not be calculated because of oscillations within each pulse due to the sparseness of the array. By contrast, double averaging of the Δf–*k* analysis and FD beamforming provided consistent results at all angles, with two distinct angles (direct: 13.8${}^{\circ }$, surface-reflected: −36${}^{\circ }$), demonstrating consistency between Figure 3 and Figure 4b. Compared with the reference angles, the maximum error of the angle estimated in Figure 5a is approximately 1${}^{\circ }$, indicating that the angle is successfully estimated.

Figure 5b shows the results of double averaging over all difference frequencies (i.e., 0–1400 Hz) used in this paper. The result of DAS beamforming is identical to that shown in Figure 5a. The two mainlobes of double averaging remained similar and distinguishable. For simulation data, the angles estimated from the Δf–*k* analysis and FD beamforming are as follows: (1) Δf–*k* analysis, 16.4${}^{\circ }$ (direct) and −36.5${}^{\circ }$ (surface-reflected); and (2) FD beamforming, 16.4${}^{\circ }$ (direct) and −35.0${}^{\circ }$ (surface-reflected). Compared with the reference angles, the maximum error in the estimation of all angles using the two approaches is 4${}^{\circ }$ (i.e., the surface-reflected path in FD beamforming). This error is believed to be caused by the angle spread due to the near-field effect on difference frequencies above 400 Hz, and the error decreased as the range increased. In addition, there is a noticeable difference in the sidelobes between Figure 5a,b. The difference between the Δf–*k* analysis and FD beamforming is based on whether the gray-shaded regions are included when double-averaged over all difference frequencies. This is the region where the grating lobes of FD beamforming are formed, and all grating lobes are included in the case of double averaging in FD beamforming. This yields the same effect as adding background noise. As a result, the sidelobe of FD beamforming is larger than that of the Δf–*k* analysis. The simulation confirmed that, although both approaches can estimate DOAs, there may be a difference in the sidelobe depending on the difference-frequency band for double averaging.

## 5. Experimental Results

**Figure 6:**
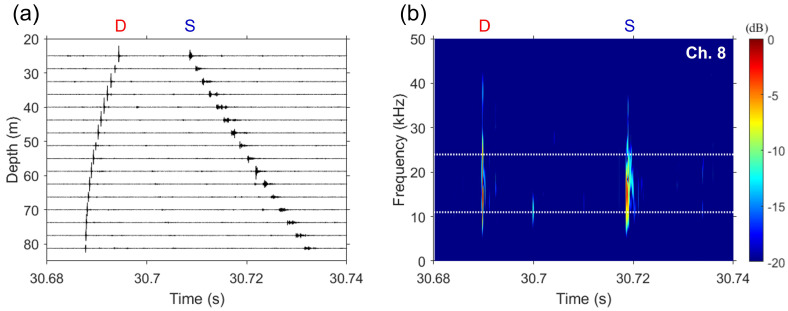
(**a**) Cracking sounds recorded by the VLA on JD 141 (JD141 06:55:30). D and S denote the direct and surface-reflected paths, respectively, separated at around 30.7 s. (**b**) Spectrogram of cracking sounds received on a single element of the VLA at a depth of 51.25 m (eighth channel). The dominant frequency band within the white dotted lines (11–24 kHz) is selected to estimate the DOAs.

To verify the proposed algorithm by using experimental data, we analyzed a set of direct and surface-reflected noises collected on JD 141 (JD141 06:55:30). Cracking sounds along the VLA are shown in Figure 6a. The direct and surface-reflected paths, which were separated at around 30.7 s, are denoted by D and S, respectively. The snapping shrimp was in the near field of the array, as evidenced by the direct path with the shape of a spherical wavefront. The surface-reflected noise was dispersive due to the rough sea surface. As an example of a cracking sound, which has a higher spectral energy at frequencies above 10 kHz [24], the spectrogram of the received signal at the middle hydrophone (i.e., 51.25 m; eighth channel) are displayed in Figure 6b. The dominant frequency band of cracking sounds was found to be 11–24 kHz (frequency band between the white dotted lines in Figure 6b) and was used to estimate the DOAs by using FD beamforming and the proposed algorithm.

**Figure 7:**
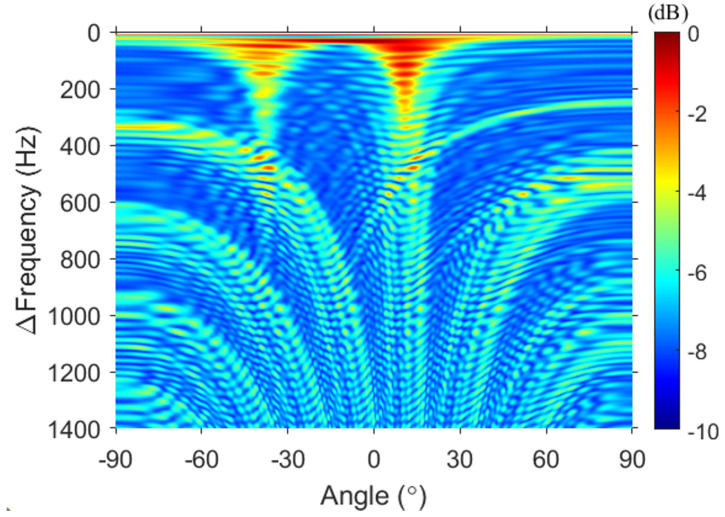
FD beamforming of experimental data. Overall, the outputs of FD beamforming of the simulation (see Figure 3) and experimental data show good agreement. In the difference frequency range below 400 Hz, two vertical lines are formed, as in the simulation. However, for a difference-frequency band higher than 400 Hz, the angle spread is more severe than that in the simulation. This more severe angle spread may have been caused by (1) the curvature of the array shape because of various factors, such as current, as well as (2) the near-field effect.

**Figure 8:**
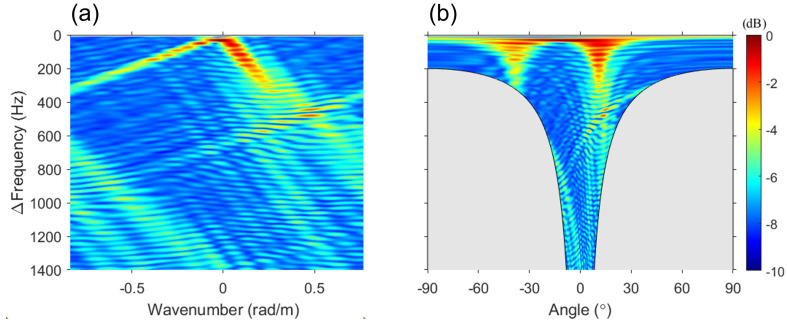
Δf–*k* analysis of experimental data. (**a**) Difference frequency–wavenumber domain and (**b**) difference frequency–angle domain. As expected from Figure 3 and Figure 7, the Δf–*k* analysis of cracking sounds is similar to that of simulation data.

**Figure 9:**
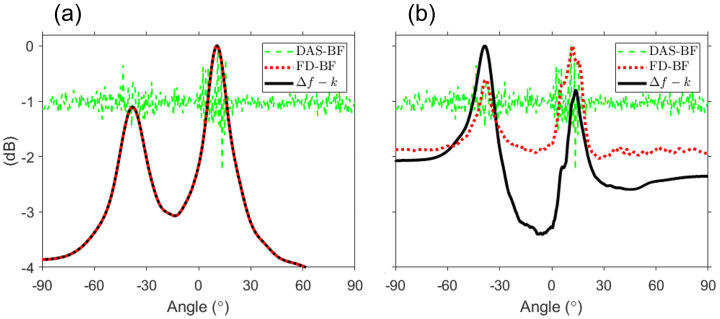
For experimental data, comparison of the Δf–*k* analysis (black solid line) with FD beamforming (red dotted line) double-averaged over 0 ≤Δf≤ (**a**) 200 Hz (design frequency) or (**b**) 1400 Hz with DAS beamforming (green dashed line). In (**b**), contrary to the expected result (**a**), not only are the sidelobes reduced but also the intensity of the direct path is lower than that of the surface-reflected path. Angle spread, which deteriorates at difference frequencies above 400 Hz, causes destructive interference. Consequently, as double-averaging the direct path includes a wider angle–spread region, the intensity is relatively lower than that of the surface-reflected path.

Through simulations, we found that the *f*–*k* analysis for the sparse vertical array configuration is featureless. As this featurelessness appears similarly in the data of FD beamforming, the *f*–*k* analysis for the snaps measured from the experiment is not displayed. Figure 7, Figure 8 and Figure 9 illustrate the results along with the experimental data in the same manner as the simulation results (see Figure 3, Figure 4 and Figure 5). Recall that averaging for $\omega_{1}$ was performed with 10-Hz intervals between 11 kHz and 22.6 kHz. Figure 7 shows the FD beamforming output as the experimental result counterpart of Figure 3. Two vertical lines appear between −60${}^{\circ }$ and 30${}^{\circ }$ in the frequency band below 400 Hz, as in the simulation; however, afterward, the two lines spread and disappeared in the frequency band. This phenomenon is believed to be caused by the array shape. In contrast to the simulation, which assumes that the array is a straight line, the array during the experiment was not straight and might have had a curvature because of various factors such as current. This curvature can potentially cause an angle spread similar to that caused due to the near-field effect, and a direct arrival through a relatively closer path will be more sensitive. Nevertheless, except for the difference in the background noise, the patterns of the main and grating lobes in Figure 3 and Figure 7 are similar, suggesting that the Δf–*k* analysis of the experimental data is similar to that of the simulation data.

Figure 8 shows the output of the Δf–*k* analysis as the counterpart of the experimental results in Figure 4. The experimental data were comparable to the simulation results, as expected from the FD beamforming output. Furthermore, the angle spread and decay caused by the abovementioned array shape were more clearly highlighted by the angle-related slope in the Δf–*k* domain (see Figure 8a). Despite the addition of background noise and the influence of the array shape compared with the simulation, the two main lines can still be clearly identified in Figure 8.

Figure 9 depicts the results of the experimental data obtained by employing double averaging, which can improve the robustness and the output of DAS beamforming. Double averaging is performed with an interval of 1 Hz starting at 0 Hz, and the upper bounds of double averaging in Figure 9a,b are 200 Hz (design frequency) and 1400 Hz, respectively. As all angles can be detected by using the Δf–*k* analysis in the difference-frequency band within the design frequency, the results of FD beamforming and Δf–*k* analysis double-averaged from 0–200 Hz coincide, as illustrated in Figure 9a. This is the expected result, which is the same as the simulation result. By contrast, data in Figure 9b, in which the upper bound of double averaging is 1400 Hz, differ from the simulation data (see Figure 5b). The intensity of the direct path is greater than that of the surface-reflected path in the simulation, as shown in Figure 5b, whereas the intensity of the surface-reflected path is greater in the Δf–*k* analysis in Figure 9b. Although not shown here, the intensity of the direct path steadily decreased when the upper bound of double averaging is gradually increased from 200–1400 Hz. This drop in the direct path intensity is expected owing to the angle spread. In contrast to the surface-reflected path, wherein the angle spread is not apparent due to detectable angle restriction, the intensity of the direct path decreased because of destructive interference caused by the angle spread. Nonetheless, we confirmed that the two mainlobes can be clearly identified and that the sidelobes, after double averaging the *f*–*k* analysis, are lower than those after FD beamforming, as in the simulation. The two peaks in Figure 9a, which are not affected by angle spread, are 10.5${}^{\circ }$ (direct) and −38${}^{\circ }$ (surface-reflected) shifted by −2${}^{\circ }$ from the angles (see Figure 5a) estimated in the simulation. Because there was an array tilt during SAVEX15 [7,24], which caused a shift in the angular axis, the angle shift between the experimental and simulation data is reasonable.

## 6. Conclusions

For a sparse vertical array configuration, the *f*–*k* analysis, which can be used to estimate the DOA of a wideband signal, has a significantly limited detection angle. Additionally, the DOA cannot be estimated due to interference if there is a multipath. To solve this problem, we proposed the Δf–*k* analysis, in which the FD concept of, utilized for sparse vertical array configuration, was adapted to the *f*–*k* analysis. The performance of the Δf–*k* analysis was verified via simulation in the SAVEX15 environment and was compared with DAS and FD beamforming. Subsequently, the cracking sounds recorded by a sparse vertical array configuration during the SAVEX15 experiment were analyzed. The Δf–*k* analysis effectively estimated the DOA of a sparse vertical array configuration, which DAS beamforming could not estimate. Additionally, analogous to the relationship between the *f*–*k* analysis and DAS beamforming, we verified that the Δf–*k* analysis is closely related to FD beamforming. The outputs of the two algorithms became identical when the difference frequency was lower than the design frequency. However, when the difference frequency was higher than the design frequency, the detectable angle of the Δf–*k* analysis was limited, resulting in reduced sidelobes in the double-averaged Δf–*k* analysis due to the filtering effect of the grating lobes induced by FD beamforming. The simulation results are consistent with the experimental data, indicating that angle estimation using the Δf–*k* analysis is feasible for sparse vertical array configuration and that the Δf–*k* analysis and FD beamforming are closely related.

## Data Availability

Not applicable.
